# Evaluating Critical Influencing Factors of Desalination by Membrane Distillation Process—Using Multi-Criteria Decision-Making

**DOI:** 10.3390/membranes11030164

**Published:** 2021-02-27

**Authors:** Ali E. Anqi, Azam A. Mohammed

**Affiliations:** Department of Mechanical Engineering, College of Engineering, King Khalid University, Abha 61421, Saudi Arabia; mazam@kku.edu.sa

**Keywords:** membrane distillation, critical influencing factors (CIFs), analytic hierarchy process (AHP), Fuzzy-AHP, multiple decision maker (MDM), derivable output

## Abstract

Water desalination by membrane distillation (MD) can be affected by a wide range of operating parameters. The present work uses combinational approach of Analytical Hierarch process (AHP) and Fuzzy Analytical Hierarchy process (Fuzzy-AHP) to identify the most important parameters in the MD desalination. Five process parameters and key-performance indicators, named derivable outputs (DOs), are considered, along with the critical factors affecting these DOs in the current study. The DOs and the critical influencing factors (CIFs) are selected based on their experimental feasibility. The investigation involves five DOs, which are liquid entry pressure, thermal power consumption, permeate quality, permeate flux, and pumping (feed circulation) power. A total of twenty-five critical influencing factor were associated with the DOs. The identification of the DOs and CIFs was based on the literature review, and further analyses were performed. Both methods, AHP and Fuzzy-AHP, determined six extremely important CIFs in the desalination MD, which are feed temperature, feed concentration, or feed salinity; feed flow rate; membrane hydrophobicity; pore size; and membrane material. Moderately important CIFs are found to be four by both methods. These common CIFs are feed solution properties, membrane thickness, feed channel geometry, and pressure difference along the feed channel. Finally, the least preferred CIFs are four common in both methods that are MD configuration, duration of test, specific heat of feed solution, and viscosity.

## 1. Introduction

Freshwater is a basic requirement globally. With continuous increase in population and the rapid exhaustion of freshwater resources, the attention is now turning towards the desalination of seawater. Even now, 11% of the total world’s population has no access to portable pure water. Although world surface comprises of 70% water, 97% of which being salty and is inaccessible to human use. Industrialization, diminishing rainfall and global warming worsened the situation further to reach a critical point wherein available pure water is also getting exhausted [1]. To meet these constant increasing water needs, seawater desalination or brackish water treatment can be helpful to face the global demand of freshwater. Abundant water supply can be obtained by seawater desalination, which includes separation of impurities, minerals, and salts to reduce the excessive salt concentration of the seawater to become portable water. However, this removal of salt from seawater is an expensive process [2].

**Figure 1:**
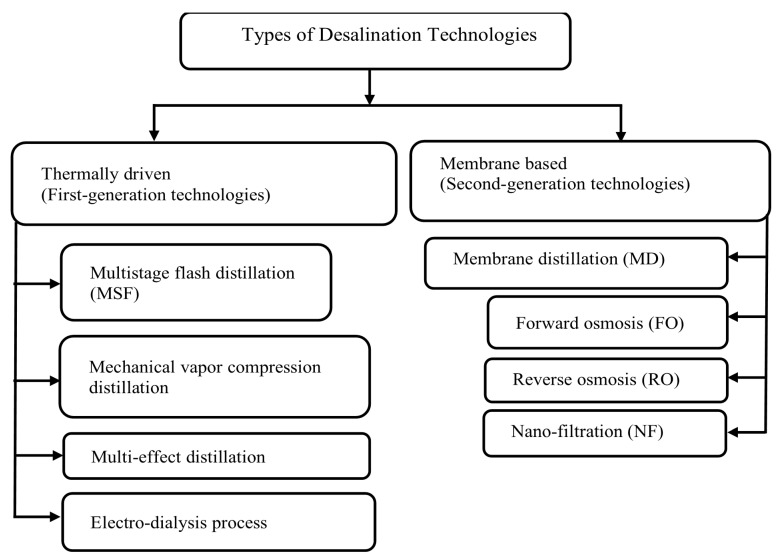
Types of desalination technologies.

The produced water by the second generation technologies (Figure 1) comes in a better quality and higher cost efficiency compared to the first generation technologies. As the salt removal capacity of membrane distillation (MD) and reverse osmosis (RO) is high, these processes are extensively studied. MD is basically a membrane thermal driven process, but RO is pressure-driven [1]. Membrane distillation (MD) is proving to be a promising technology. It basically uses thermal energy for the separation of water. In this process, only water vapor can pass through the pores of hydrophobic microporous membrane. In comparison with other desalination methods, MD exhibits great advantages, such as energy utilization, low operating temperature, and high salt rejection rate. The hydrostatic pressure is also less, as of what is observed in the RO process. Hence, MD could be a competitive process once fully commercialized. Considering these conclusions, affordable materials can be deployed in MD. Plastic can be used to reduce corrosion issues [3].

The process of MD can be classified into four main types of configurations [4,5]:

1. Direct Contact Membrane Distillation (DCMD): The membrane in this method is directly in contact with the hot feed solution and cold permeate stream [6]. Difference in temperature and partial pressure between the two sides across the membrane causes the molecules of water to evaporate at the interface of the membrane and the hot feed stream. Condensation of the formed vapor takes place at the interface of the membrane and the cold stream, after passing through the membrane pores [7].
2. Air Gap Membrane Distillation (AGMD): In this type of MD, an air gap is insinuated between the membrane and the condensation surface where the generated water vapor condenses. The other side of the condensation surface is cooled by a cold stream [8].
3. Sweep Gas Membrane Distillation (SGMD): In this method of MD, a carrier in the form of a cold inert gas is used to sweep the generated water vapor formed on the permeate membrane side [9]. The condensation takes place in a separate chamber outward from the membrane module.
4. Vacuum Membrane Distillation (VMD): A vacuum pressure is applied to increase the partial pressure difference across the membrane to maximize the driving force of the water evaporation, wherein a vacuum pump is used to apply vacuum on the permeate side of the membrane [10]. As the saturation vapor pressure is higher than the applied vacuum pressure, the evaporation rate is augmented. The condensation of the produced water vapor takes place in a condenser outside the membrane module [11].

Some of the benefits of MD can be listed as [12]:

- MD process is a reliable alternative to other desalination methods as it functions at reduced pressure leading to decreased equipment cost and improved safety process.
- MD is an environmentally friendly and cost-efficient process, as it can utilizes energy from solar sources and even the waste heat from other processes.
- MD is successfully being used in food industry for the concentration of juices and milk.
- MD has low organic fouling, low energy cost, and the absence of limitations caused by osmotic pressure effect.

Multi-Criteria Decision-Making (MCDM) is a process of finding the best set of alternatives by comparing them to get the best results. Analytical Hierarchy process (AHP) is a type of (MCDM) tool. AHP is mathematically structured to help in solving multicriteria problems. This method requires conviction in assessing the importance of each decision bases against others using crisp numeric to estimate better. Fuzzy concept improves the old AHP method in capturing the fuzziness of multiple decision-makers by using linguistic terms, like extremely preferred, moderately preferred, etc. Fuzzy-AHP is the ranking structure in which a relative comparison is estimated using fuzzy membership [13]. Triangular fuzzy numbers can be used in preparing pairwise comparison matrix using Fuzzy-AHP.

In the last decade, different studies have been conducted in the field of water treatment and desalination by using MCDM. Srdjevic et al. [14] applied AHP to evaluate four wastewater treatment methods to treat water from colored metals industry. The study found that the biological treatment method was the optimal compared to the other three methods (evaporation, chemical, and separation). Manekar et al. [15] utilized Fuzzy-AHP to optimize the water pretreatment module for the produced water in the textile industry, and they reported that Fuzzy-AHP is an effective optimization method. Chamblas and Pradenas [16] deployed three MCDM methodologies (AHP, ELimination Et Choice Translating REality (ELECTRE), and Technique for Order of Preference by Similarity to Ideal Solution (TOPSIS)) to select optimum seawater desalination method. The study examined six different desalination methods and reported membrane technology as superior. Al-Araidah et al. [17] employed Fuzzy-AHP to inspect technical and nontechnical criteria in the selection of RO membrane. The inspection concluded that the highest technical and non-technical criteria were salt rejection and cost, respectively. Talaeipour et al. [18] applied AHP to find the optimum desalination method for groundwater by comparing nano-filtration (NF), RO and hybrid NF/RO. The study found that the best performance was the hybrid NF/RO. To the best knowledge of the authors, the application of MCDM has never been performed to identify the most critical parameters for desalination by membrane distillation.

The aim of this study is to estimate and prioritize the critical influencing factors (CIFs) of MD process using MCDM approach. The study uses AHP and Fuzzy-AHP to evaluate the effect level of the critical factors.

## 2. Methodology

Some important factors have come out in this area by reviewing the literature. Based on the attempts of different researchers and their study on different factors which were influencing the process of MD, five types of derivable outputs are considered for evaluation. Optimization of these derivable output will help in estimating the rank of each critical influencing factor. The five derivable outputs which affect the process of membrane distillation are thermal power, liquid entry pressure, rejection rate, pumping power, and permeate flux. Critical influencing factors of MD are identified based on the following steps:

1. Detailed analysis of literature on membrane technologies and effect of different factors influencing membrane distillation.
2. Identification of critical factors which influence the performance of the MD process.

**Figure 2:**
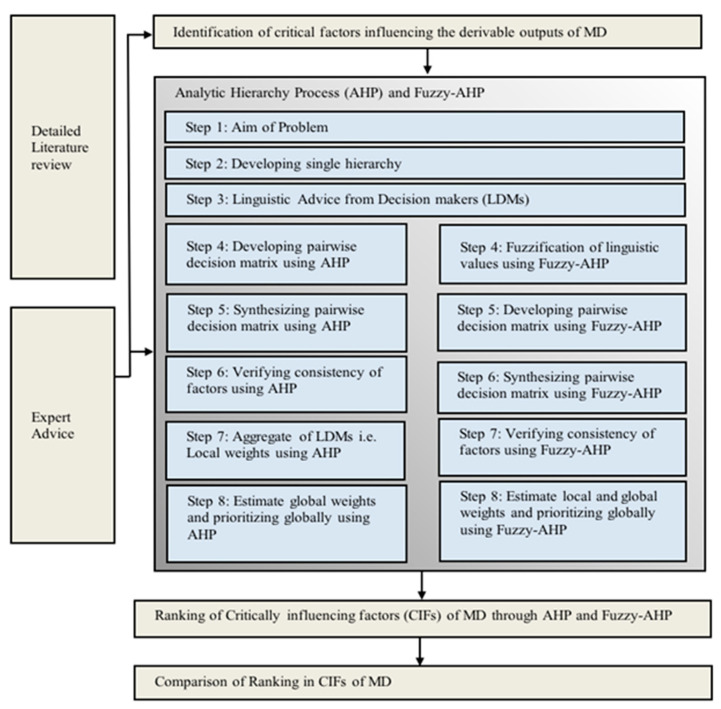
Framework of critical influencing factors of membrane distillation (MD) based on Fuzzy-Analytical Hierarch process (AHP)-multiple decision-maker (MDM).

Based on the above steps, a framework was made to select CIFs as shown in Figure 2. These CIFs of MD are used to improve the derivable outputs (DOs) based on their weightages and prioritization. Mixed approach of Fuzzy-AHP and AHP-multiple decision-maker (MDM) are utilized in attaining the subsequent priorities of CIFs grouped together. Detailed analysis of literature was helpful in finalizing the five derivable outputs from the existing seven derivable outputs. The derivable outputs that were neglected in this research are, temperature polarization coefficient and concentration polarization coefficient. Table 1 describes the derivable outputs which are further categorized into CIFs, based on which the weightages were assigned and were later prioritized.

Two methodologies, namely AHP-MDM and Fuzzy-AHP, are presented in this study. AHP is used in solving complex problems which require systematic decision-making. It utilizes multi-level hierarchy of goal outputs, as well as sub-critical factors, whereas Fuzzy-AHP uses fuzzy set theory and the extension principle based on goal-oriented application. To avoid vagueness, multiple decision-makers are incorporated hence fruitful results from AHP-MDM are obtained.

### 2.1. Analytic Hierarchy Process (AHP) Theory

Analytic Hierarchy process (AHP) is the process of solving multi-level and multi-criteria problems. It utilizes pairwise judgmental matrix obtained from the geometric mean of multiple decision-makers (MDM). Vast experience of decision-maker is used to frame the linguistic pair-wise decision matrix from Saaty’s nine-point scale as shown in Table S1 in the Supplementary Materials [170]. AHP process is based on two main objectives, i.e., expert knowledge and experience in obtaining final opinion. Final opinion is taken and assigned linguistic terms to these opinions which help in framing the pairwise comparison matrix of each expert. Biases in decision that can be avoided by considering multiple experts’ opinions are termed as multiple decision-makers (MDM). By taking the geometric mean of all the pairwise matrices and synthesizing, this may lead to highly accurate single AHP-MDM matrix.

The basic steps of AHP are illustrated here:

Step 1: Derivable outputs (DOs) and (CIFs) of MD are combined to form a pair-wise comparison matrix ‘*C*’. This matrix ‘*C*’ is the decision matrix and is prepared by using crisp numeric values of Saaty’s nine-point scale, as shown in Table S1 in the Supplementary Materials. Matrix ‘*C*’ is the preference level of each element with respect to the other element. It can be said that element *C_ij_* is preference of *i*th element with respect to *j*th in terms of preference level.

(1) $$C=\left[\begin{array}{cccc} C_{11} & C_{12} & \ldots & C_{1j}\\ C_{21} & C_{22} & \cdots & C_{2j}\\ \vdots & \vdots & \vdots & \vdots \\ C_{i1} & C_{i2} & \ldots & C_{ij} \end{array}\right]$$

Step 2: From each linguistic expert advice, pairwise comparison matrix ‘*C*’ is developed to form each decision matrix (DM). Then a geometric mean is obtained for each DM, and the geometric mean is normalized to obtain the priority vector.

Step 3: The elements from the ‘*C*’ matrix are utilized to obtain the summation of the product of the sum of the column vector of each element in the DM, pair-wise DM with the values of priority vector in the corresponding row to obtain the principal Eigen values as given below:

(2) $$\lambda_{Eigen}=\overset{k}{\underset{i,j}{\sum }}C_{i}P_{j}$$

where $\lambda_{Eigen}$ is the principal Eigen value, $C_{i}$ is the sum of the column vector, and $P_{j}$ is the priority vector values of each row of the DM.

Step 4: Any AHP process is subjected to consistency check. It decides the consistency of the decision-maker as per derived decision matrix ‘*C*’. The consistency index is estimated by using Equation (3) as shown [17]:

(3) $$\left(C.I\right)=\frac{\left(\lambda_{Eigen}-N\right)}{\left(N-1\right)}$$

where C.I is called consistency index, N is the total number of elements in each matrix.

Step 5: To estimate final consistency ratio it is mandatory to calculate random index which is given by Equation (4) as shown:

(4) $$R.I=\frac{1.98\;\left(N-2\right)}{N}$$

where R.I is called random index.

Step 6: It is mandatory to have consistency ratio less than 10%, only then the decision matrix is said to be satisfied. If the consistency ratio is more than 10% then it is subjected to further revision. This consistency ratio is calculated by Equation (5) as shown:

(5) $$C.R=\frac{C.I}{R.I}$$

where C.R is called consistency ratio.

Step 7: From the decision matrix ‘*C*’, the fuzzy numerical value table is generated, as shown in Table S2 in the Supplementary Materials. This fuzzy linguistic scale is used to obtain the pairwise comparison matrix (*M_i_*, *i* = 1, 2, …, *n*) for main derivable output of MD. Table S1 in the Supplementary Materials can be used in assigning weightages to these matrices. The Eigen values, C.I, R.I, and C.R, are estimated as in steps 2 to 6, respectively.

### 2.2. Fuzzy-AHP Theory

Fuzzy-AHP is the application of extension principal together with fuzzy set theory. This is utilized to avoid inaccuracies and biases during decision-making. Fuzzy-AHP is preferred over AHP due to the removal of manual judgment errors. Fuzzy-AHP is also more efficient because realistic decisions are attained from the given set of criteria’s and alternatives. Basic steps of a Fuzzy-AHP are to define the goal and create a pairwise comparison matrix to check the consistency of the problem. Fuzzy triangular number can be set to attain the weightages and ranking. Following section describes the Fuzzy set theory and application of this in fuzzy state. The Fuzzy-AHP set theory helps the decision-maker in designing robust pairwise matrix. By selecting crisp numeric values from decision-maker, biases may inculcate leading to misleading alternatives. To avoid such vagueness in values a set of fuzzy triangular number (*p*, *q*, *r*) and trapezoidal fuzzy number (*p*, *q*, *r*, *s*) are utilized to obtain the pair-wise decision matrix. Figure S2 in the Supplementary Materials represents the typical form of fuzzy triangular number.

Arithmetic operations can be carried out by using two sets of fuzzy triangular numbers. Fuzzy triangular numbers are represented by $\tilde{A_{1}}\;and\;\tilde{A_{2}}$ as $\left(p_{1},q_{1},r_{1}\right)$ and $\left(p_{2},q_{2},r_{2}\right)$, respectively. Fuzzy triangular numbers are useful in obtaining the information and helpful in attaining information of vagueness and uncertainty. Various types of arithmetic operations carried out can be represented by the following equations. Only two fuzzy triangular numbers are used to undergo any arithmetic operation (Figure S2 in the Supplementary Materials).

(6) $$\tilde{A_{1}}⊕\tilde{A_{2}}=\left(p_{1}+p_{2},q_{1}+q_{2},r_{1}+r_{2}\right)$$

(7) $$\tilde{A_{1}}⊖\tilde{A_{2}}=\left(p_{1}-p_{2},q_{1}-q_{2},r_{1}-r_{2}\right)$$

(8) $$\tilde{A_{1}}⊗\tilde{A_{2}}=\left(p_{1}p_{2},q_{1}q_{2},r_{1}r_{2}\right),$$

(9) $$\lambda ⊗\tilde{A_{1}}=\left(\lambda p_{1},\lambda q_{1},\lambda r_{1}\right)\;where\;\lambda >0,\;\lambda \in R$$

(10) $${\tilde{A_{1}}}^{-1}=\left(\frac{1}{r_{1}},\;\frac{1}{q_{1}},\;\frac{1}{p_{1}}\right)$$

Principle of Extension analysis:

Comparison of two fuzzy triangular numbers is done by extent analysis. Goal and objective are two sets given by X = {x_1_, x_2_, … x*_n_*} and Y = {Y_1_, Y_2_, … Y*_n_*}, respectively. Extent analysis for each goal is carried out by extent principle, so ‘*m*’ extent values are as follows:

(11) $$A_{gi}^{1},\;A_{gi}^{2},\ldots \;A_{gi}^{m},\;i=1,\;2,\ldots ,\;n$$

(12) $$\mathrm{where}\;A_{gi}^{j}\left(j=1,\;2,\ldots ,\;n\right)$$

Equation (12) represents fuzzy triangular numbers and are represented by (*p*, *q*, *r*)

**Figure 3:**
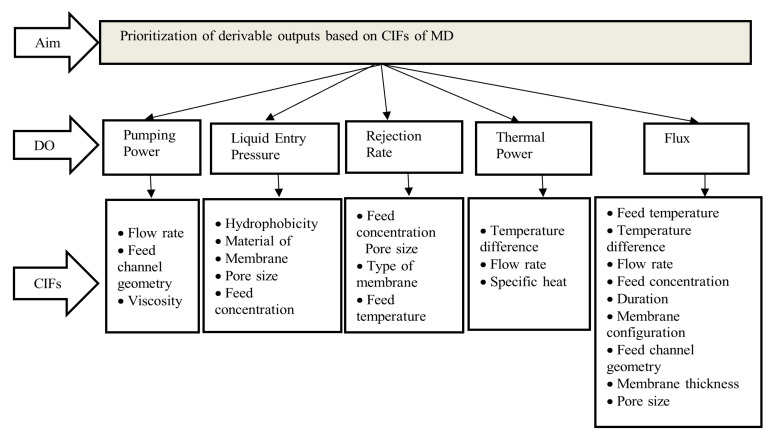
Multi-Criteria Decision-Making (MCDM) model for output-based analysis of MD.

Step 1: Developing structural network for the above goal:

Membrane distillation techniques are grouped into number of multiple levels which consists of DOs and CIFs. Based on the decision-maker, structural network is verified and arranged as per hierarchical order for ranking. The DO and CIFs are placed as per priorities shown in Figure 3.

Step 2: Generating pairwise comparison matrices for DOs and CIFs of membrane distillation:

As per the decision-makers, a pairwise comparison matrix was developed for both DOs and CIFs. In the matrix, fuzzy triangular numbers were used in developing relationship between each other elements of matrices.

Step 3: Attaining value of Fuzzy extent:

(13) $$F_{i}=\overset{m}{\underset{j=1}{\sum }}A_{gi}^{j}⊗{\left[\sum_{i=1}^{n}\sum_{j=1}^{m}A_{g\;i}^{j}\right]}^{-1}$$

Using summation of fuzzy triangular number, *m* extent values $\sum_{j=1}^{m}A_{g\;i}^{j}$ are attained:

(14) $$\sum_{j=1}^{m}A_{g\;i}^{j}=\left(\sum_{j=1}^{m}p_{j},\;\sum_{j=1}^{m}q_{j},\;\sum_{j=1}^{m}r_{j}\right)$$

(15) $${\left[\sum_{i=1}^{n}\sum_{j=1}^{m}A_{g\;i}^{j}\right]}^{-1}$$

where Equations (14) and (15) represent summation of following equation:

(16) $$A_{g\;i}^{j}\;\left(j=1,\;2,\ldots ,\;m\right)$$

which is calculated as follows:

(17) $$\sum_{i=1}^{n}\sum_{j=1}^{m}A_{g\;i}^{j}=\left(\sum_{j=1}^{m}p_{j},\;\sum_{j=1}^{m}q_{j},\;\sum_{j=1}^{m}r_{j}\right)$$

Inverse of Equation (17) will be

(18) $${\left[\sum_{i=1}^{n}\sum_{j=1}^{m}A_{g\;i}^{j}\right]}^{-1}=\left(\frac{1}{\sum_{i=1}^{n}r_{i}},\;\frac{1}{\sum_{i=1}^{n}q_{i}},\;\frac{1}{\sum_{i=1}^{n}p_{i}}\right)$$

Step 4: Degree of supremacy from the possibility of two fuzzy triangular numbers are given by the following relation [13,17]:

(19) $$A_{2}=\left(p_{2},\;q_{2},\;r_{2}\right)\geq A_{1}=\left(p_{1},\;q_{1},\;r_{1}\right)$$

Supremacy is given by the relation

(20) $$V\left(A_{2}\geq A_{1}\right)=\sup\left[\min\left(\mu_{A1}\left(x\right),\;\mu_{A2}\left(y\right)\right)\right],\;y\geq x$$

Transforming Equation (20) gives Equations (21) and (22):

(21) $$V\left(A_{2}\geq A_{1}\right)=hgt\;\left(A_{2}\cap A_{1}\right)=\mu_{A2}\left(f\right)$$

(22) $$\mu_{A2}\left(f\right)=\{\begin{array}{cc} 0 & if\;q_{2}\geq q_{1}\\ 1 & if\;p_{1}\geq r_{2}\\ \frac{\left(p_{1}-r_{2}\right)}{\left(q_{2}-r_{2})-(q_{1}-r_{1}\right)} & otherwise \end{array}$$

As there are multiple decision-makers involved, there will be ‘*K*’ matrices for ‘*K*’ number of decision-makers, resulting in ‘*n*’ number of elements

(23) $$\tilde{M_{K}}=\left\{\overset{ˇ}{p_{ijk}}\right\},$$

(24) $$\mathrm{where}\;\tilde{M_{K}}=\overset{ˇ}{p_{ijk}}=\left(p_{ijk},q_{ijk},r_{ijk}\right)$$

Equation (24) represents relative importance of *i*th to *j*th element with respect to ‘*K*’ decision-makers. Hence, Equations (25)–(27) represent the aggregate.

(25) $$p_{ij}=\min\left(p_{ijk}\right),\;k=1,\;2,\ldots ,\;k,$$

(26) $$q_{ij}=\sqrt[{k}]{\overset{k}{\underset{k=1}{a}}q_{ijk}}$$

(27) $$r_{ij}=\max\left(r_{ijk}\right),\;k=1,\;2,\ldots ,\;k$$

The two fuzzy triangular numbers $A_{2}=\left(p_{2},\;q_{2},\;r_{2}\right),\;A_{1}=\left(p_{1},\;q_{1},\;r_{1}\right)$ intersects at ‘F’, as shown in Figure S2 in the Supplementary Materials. From the highest intersection between two fuzzy numbers, $\mu A_{1}$ and $\mu A_{2}$ ordinate, *q* is obtained. Hence, $A_{1}$ and $A_{2}$ can be calculated by the following equations:

(28) $$V\left(A_{1}\geq A_{2}\right)\;\mathrm{and}\;V\left(A_{2}\geq A_{1}\right)$$

Step 5: Attain the degree of possibility for convex fuzzy number which must be greater than ‘*k*’ convex:

(29) $$A_{1}\left(i=1,\;2,\ldots ,\;k\right)$$

Equation (29) represents fuzzy number, which is derived as

(30) $$V\left(A\geq A_{1},A_{2},\ldots ,A_{K}\right)=V[\left(A\geq A_{1})\;and\;(A\geq A_{2}\;and\;\ldots and\;\left(A\geq A_{K}\right)\right)]\;=\min V\left(A\geq A_{i}\right),\;i=1,\;2,\ldots ,\;k$$

Hence,

(31) $$d\left(a_{i}\right)=\min V\left(F_{i}\geq F_{K}\right)\;for\;k=1,\;2,\ldots ,\;m;\;k\neq i$$

Weight vector is obtained by:

(32) $$W^{\prime }={\left(d^{\prime }\left(a_{1}\right),\;d^{\prime }\left(a_{2}\right),\ldots ,\;d^{\prime }\left(a_{n}\right)\right)}^{T}$$

such that $a_{i}\left(i=1,\;2,\ldots ,\;n\right)$ has ‘*n*’ number of elements.

Step 6: Evaluate normalized weight vectors.

Normalized weight vectors are estimated using Equation (33):

(33) $$W={\left(d\left(a_{1}\right),\;d\left(a_{2}\right),\ldots ,\;d\left(a_{n}\right)\right)}^{T}$$

where ‘*W*’ is a numeric value.

Step 7: Estimating the score of each CIF dimension and prioritization.

The overall priority weightages of each DO and its CIFs are estimated by taking the product of global weight and local weight. The global weightages of DO and CIFs are arranged in decreasing order to give the rank.

A mixed approach of AHP-MDM and Fuzzy-AHP has been used in deriving and ranking CIFs of MD. AHP-MDM provides a synthetic assessment from linguistic decision-makers (LDM), whereas Fuzzy-AHP helps in removing biases present in the decision-making. Decision-makers play a critical role in qualitative assessment. Naveed et al. [13] used three and five decision-makers in AHP and Fuzzy-AHP and found having three decision-makers gave more accurate results whereas five decision-makers showed vagueness. Based on the literature review [13,17,18,170,171], three LDMs with more than five years’ experience in membrane distillation were considered. One of the decision-makers is from the industrial field (desalination plant), and two are from the academic field. Apart from the core engagements, they also had long exposure in the field of membrane distillation. The LDMs were convinced to unite for the academic and research cause for which they agreed without any reservation whatsoever. A detailed review of the literature was carried out for identifying CIFs of MD. Five main derivable outputs were identified, which are pumping power, liquid entry pressure, rejection rate, thermal power, and flux. Figure 3 shows the framework for evaluating and prioritizing the derivable outputs based on CIFs of MD.

Three linguistic decision-makers (LDMs), i.e., LDM1, LDM2, and LDM3, assessed five dimensions that are given in Table 2, Table 3 and Table 4. Table 2 is the linguistic decision matrix obtained from the decision-maker-1 (LDM-1). As per the preferences of the decision-maker, weightages of dimensional output were estimated by using AHP process. The weightages obtained suggest that decision-maker-1 feels permeate flux is the most important output, whereas thermal power and pumping power were rated as the least preferred. Liquid entry pressure and rejection rate were rated almost equally after flux by decision-maker-1. Table 3 is also linguistic decision matrix obtained from decision-maker-2 (LDM-2). Though flux was most preferred output and pumping power was given as the least preferred output by decision-maker-2, the order of preference as per LDM2 were flux, rejection rate, liquid entry pressure, thermal power, and pumping power, respectively. The decision matrix from linguistic decision by decision-maker-3 (LDM-3) is shown in Table 4. As per the pairwise matrix of LDM3, weightages were estimated using AHP and found to have almost the same sequence as that of other decision-makers as flux, liquid entry pressure, rejection rate, thermal power, and pumping power, respectively.

## 3. Results and Discussions

Table 5 is obtained after synthesizing the results of the three decision-makers. Weights from AHP-MDM are subjective weights, as it purely depends on the decision-maker. Decision-makers have utilized the experience combined with literature to decide the preference of each output with respect to the others. To avoid vagueness in weightages, geometric mean of Table 2, Table 3 and Table 4 is carried out over arithmetic mean as the former is not characterized by reciprocity. This is to have more consistency in the final weightages. The order of the final weightages is: ‘flux’, followed by ‘rejection rate’, then ‘liquid entry pressure’ which is continued by ‘thermal power’, and, finally, ‘pumping power’. Table 5 provides synthetic results which are obtained from AHP-MDM, which is less biased than the AHP analysis of each LDM’s.

As the next step is to achieve the aggregated value from the multiple decision-makers to single relevant matrix, which is composite weightages of all the CIFs, as shown in Table 6. Geometric mean was again applied to weightages obtained from all decision-makers, to obtain a single value. This is repeated for each DO and its corresponding factors obtained after aggregation. This gives the local weights of each factor. The product of these local weights and weightages obtained in Table 5 were utilized in attaining the global weights of each CIF. As per the decreasing order of these global weights, the ranks were estimated as shown in Table 6.

Weightages estimated by using Fuzzy-AHP process are represented in Table 7. The triangular fuzzy number scale is used to create the pair-wise matrix in Fuzzy-AHP process. As the pairwise matrix obtained in Fuzzy-AHP consists of fuzzified numbers, synthesizing this pairwise fuzzy numbers was done as per steps mentioned in the equations 14 through 33. The weightages which consist of fuzzy number are de-fuzzified and normalized to find the final weightages shown in Table 7. Here, the weightages are similar to the ones in Table 5, which show the maximum weightage is given to ‘flux’, then ‘rejection rate’, followed by ‘liquid entry pressure’, and then ‘pumping power’, followed by ‘thermal power’, respectively.

The aggregate values of all the CIFs obtained by using Fuzzy-AHP are listed in Table 8. Fuzzified pairwise matrix for DOs and their corresponding factors is performed to estimate the weightages. The product of these local weightages with the weightages obtained in Table 7 leads to global weightages. Table 8 consists of aggregate weightages, global weightages, and the values of local weightages of all the CIFs. Therefore, Table 8 is termed as composite weightages of CIFs using Fuzzy-AHP. Accordingly, the global weightages and rankings are estimated.

**Figure 4:**
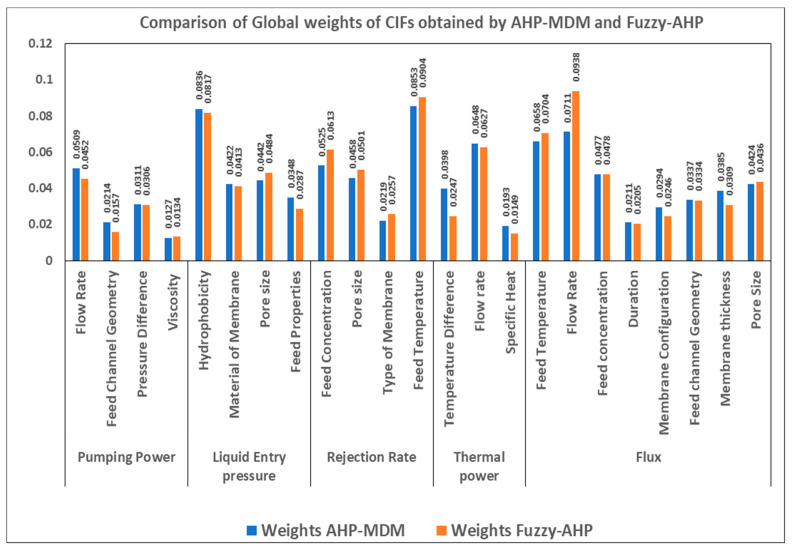
Comparison of weights of CIFs of membrane distillation obtained using AHP-MDM and Fuzzy-AHP.

**Figure 5:**
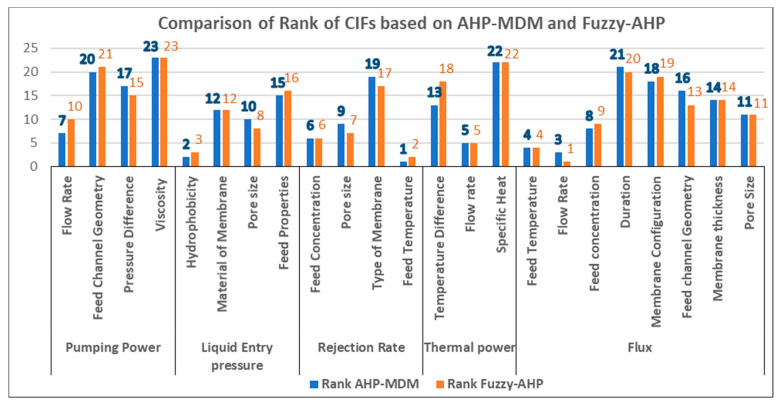
Comparison of ranks of CIFs of membrane distillation obtained using AHP-MDM and Fuzzy-AHP.

Figure 4 illustrates the criteria of weightages obtained for all the CIFs using both AHP-MDM and Fuzzy-AHP. It represents the weightages of all the 23 CIFs using both AHP-MDM and Fuzzy-AHP. In Figure 4, the values of weightages estimated by using AHP-MDM and Fuzzy-AHP are not the same. The weightage of flow rate is 0.0938, attained highest value, followed by feed temperature, which is 0.0904, and then hydrophobicity, with a value of 0.0817, as estimated using Fuzzy-AHP, whereas the weightage of feed temperature with value of 0.0853 was highest, followed by hydrophobicity with a value of 0.0836, and then followed by 0.0711 for flow rate, as calculated using AHP-MDM. To have a clear picture and understanding of both methods, graphs representing the ranking of both methods are plotted in Figure 5.

Figure 5 focuses on prioritizing all the CIFs based on both AHP-MDM and Fuzzy-AHP processes. As per the ranks, it is evident that flow rate is the most critical factor that influence flux. The flux has the highest value of weightage estimated using both AHP-MDM and Fuzzy-AHP. The flux is greatly influenced by flow rate followed by feed temperature. Flow rate of flux is ranked 1, followed by feed temperature, which is sub-factor of rejection rate, and then continued by hydrophobicity, which influences liquid entry pressure, according Fuzzy-AHP. The trend changes slightly when compared to AHP-MDM. As in AHP-MDM, feed temperature, which is subset of rejection rate, is ranked 1, followed by hydrophobicity, a subset of liquid entry pressure, and flow rate, which is a subset of flux ranked 3. Though viscosity is a sub-factor of pumping power, it is ranked last, preceded by specific heat, which is a subset of thermal power, maintaining the same ranks by both methods, AHP-MDM and Fuzzy-AHP. Obviously, from Figure 4 and Figure 5, there are critical factors which have influence on two or three different DOs. For example, flow rate is the most important factor that affects thermal power, pumping power, and the flux. This repetitiveness and complexity can be resolved by estimating the summation of these recurring factors namely flow rate, feed channel geometry, feed temperature, pore size, and feed concentration. The weightages were evaluated only for the CIFs by avoiding repetitiveness, as shown in Table 8. In practical terms, there are only 16 factors, which are called the CIFs, that affect different outputs of MD.

To have a broader view, Table 9 has been synthesized to rank and classify the CIFs that affect the MD process. By deploying both AHP and Fuzzy-AHP, the classification includes three groups (‘extremely important’, ‘moderately important’, and ‘least important’). From Table 9, it can be said that ranks 1 to 7 attained from AHP-MDM and Fuzzy-AHP are undisputable. The CIFs that are extremely important remain the same by both methods, AHP-MDM and Fuzzy-AHP. In case of AHP-MDM, temperature difference is considered as moderately important factor, whereas estimation by Fuzzy-AHP suggests that temperature difference is least important. Similarly, the type of membrane is considered to be least important when the value of weightage is estimated using AHP-MDM, but it is considered moderately important in case of Fuzzy-AHP analysis. Based on these results, we may recommend focusing more research on the extremely important and moderately important CIFs to improve MD desalination performance.

## 4. Conclusions

In this work, an attempt was made to prioritize the derivable outputs based on CIFs of desalination by MD. AHP and Fuzzy-AHP methods of multi-criteria decision-making were deployed to prioritize DOs and CIFs. The CIFs influencing the DOs are determined based on exclusive literature review and expert’s opinions. The study revealed that permeate flux was the major DO and followed by liquid entry pressure, while the least influencing DO was thermal power and pumping power. From the weightages obtained by both methods, the extremely important CIFs were grouped together, as flow rate, feed temperature, pore size, hydrophobicity, and feed concentration. The moderately important CIFs are material of membrane, membrane thickness, pressure difference, feed properties, and type of membrane. The least important CIFs are viscosity, specific heat, duration, temperature difference, and membrane configuration.

## Figures and Tables

**Table 1:** Derivable output and critical influencing factor (CIF) of MD.

Derivable Outputs	Critically Influencing Factors	References
Pumping Power	Flow rate/Reynold’s number	[10,19,20,21,22,23,24,25,26,27,28,29,30,31,32]
	Feed channel geometry	[10,22,23,24,33,34,35,36,37,38,39,40]
	Pressure difference along the feed channel	[21,22,33,41,42,43,44,45,46,47,48,49,50,51,52,53,54,55,56,57,58,59]
	Feed solution properties (like viscosity of liquid which is dependent on temperature and concentration of liquid)	[22,24,31,35,60,61,62,63]
Liquid entry pressure	Hydrophobicity of the membrane (like Contact angle)	[8,11,21,23,24,27,28,29,30,45,47,48,49,57,63,64,65,66,67,68,69,70,71,72,73,74,75,76,77,78,79,80,81,82,83,84,85,86,87,88,89,90,91,92,93,94,95,96,97,98,99,100]
	Material of membrane	[1,8,22,29,33,35,40,92,93,94,100,101,102,103,104,105,106,107,108,109,110,111,112,113,114,115,116]
	Pore size	[22,27,28,30,31,33,35,39,41,44,50,57,64,70,88,89,94,100,117,118,119,120,121,122,123,124,125,126,127]
	Feed solution properties, like concentration and surface tension of the feed	[23,24,29,30,35,48,60,63,112,127,128,129]
Rejection Rate	Feed Concentration	[12,28,29,30,33,39,43,45,48,52,54,59,60,63,70,73,86,88,89,100,119,123,127,129,130,131,132,133,134,135,136,137,138,139,140,141]
	Pore size of membrane	[12,21,22,27,28,30,31,33,35,39,41,44,50,57,64,70,88,89,94,100,117,118,119,120,121,122,123,124,125,130]
	Type of membrane	[1,8,22,26,33,37,39,45,98,99,100,107,108,109,110,111,112,113,114,115,116,117,118,119,120,121,122,123]
	Feed temperature	[22,23,27,33,39,41,43,46,48,51,52,54,57,59,60,68,70,86,88,89,90,100,118,119,122,123,126,130,138,139,140,141,142,143,144,145,146,147,148,149,150]
Thermal Power	Temperature Difference (between the inlet and at the outlet of the feed channel)	[12,19,21,22,33,34,39,43,52,60,119,138,145,146,149,150,151,152,153]
	Flow rate/Reynold’s number	[10,20,21,22,24,25,26,27,28,29,30,31,32,151,154,155,156,157]
	Specific heat of feed liquid	[19,22,35,73,118,151,152,158]
Flux	Feed temperature (Temperature of the tank)	[19,21,22,23,33,39,41,43,45,46,47,48,51,52,54,57,59,60,68,70,73,86,88,89,90,100,118,119,122,123,126,127,130,133,138,139,140,141,142,143,144,145,146,147,148,149,150,151,152,159,160,161]
	Flow rate/Reynold’s number	[4,10,20,21,22,24,25,26,27,28,29,30,31,32,33,34,35,36,47,50,53,57,79,94,137,140,162,163,164,165,166]
	Feed Concentration	[12,28,29,33,39,43,45,48,52,54,59,60,63,70,73,86,88,89,100,118,122,123,127,129,131,132,133,134,135,136,137,139,140,141]
	Duration of the experiment	[22,28,29,30,31,50,80,130,164,165,166,167]
	Membrane Configuration (Air gap, VMD, etc.)	[5,33,34,40,42,43,44,45,46,47,48,49,70,106,114,116,119,123,168]
	Feed channel geometry	[8,10,19,24,33,35,40,103,104,162]
	Type of membrane (varying thickness)	[22,24,28,30,34,35,36,37,38,39,64,89,94,117,121,124,126,130]
	Type of membrane (varying pore size)	[4,22,30,31,33,35,39,41,44,50,64,70,88,89,94,100,117,118,119,120,121,122,123,124,125,159,169]

**Table 2:** Pairwise comparison of derivable outputs of MD using AHP-MDM by linguistic decision-maker (LDM)1.

	Pumping Power	Liquid Entry Pressure	Rejection Rate	Thermal Power	Flux	Weightages
Pumping Power	1.00	0.50	0.33	3.00	0.33	0.1280135
Liquid Entry pressure	2.00	1.00	2.00	0.50	0.50	0.1929312
Rejection Rate	3.00	0.50	1.00	2.00	0.50	0.1894944
Thermal Power	0.33	2.00	0.50	1.00	0.20	0.1241332
Flux	3.00	2.00	2.00	5.00	1.00	0.3654277

**Table 3:** Pairwise comparison of derivable outputs of MD using AHP-MDM by LDM2.

	Pumping Power	Liquid Entry Pressure	Rejection Rate	Thermal Power	Flux	Weightages
Pumping Power	1.00	0.33	0.33	2.00	0.33	0.103005549
Liquid Entry pressure	3.00	1.00	2.00	0.50	0.50	0.210475692
Rejection Rate	3.00	0.50	1.00	4.00	0.50	0.218381505
Thermal Power	0.50	2.00	0.25	1.00	0.25	0.123796605
Flux	3.00	2.00	2.00	4.00	1.00	0.344340649

**Table 4:** Pairwise comparison of derivable outputs of MD using AHP-MDM by LDM3.

	Pumping Power	Liquid Entry Pressure	Rejection Rate	Thermal Power	Flux	Weightages
Pumping Power	1.00	0.25	0.33	2.00	0.50	0.118000588
Liquid Entry pressure	4.00	1.00	2.00	0.50	0.33	0.212988213
Rejection Rate	3.00	0.50	1.00	3.00	0.50	0.208436462
Thermal Power	0.50	2.00	0.25	1.00	0.33	0.123790924
Flux	2.00	3.00	2.00	3.00	1.00	0.336783813

**Table 5:** Synthesized results of derivable outputs of MD using AHP-MDM.

	Pumping Power	Liquid Entry Pressure	Rejection Rate	Thermal Power	Flux	Weightages
Pumping Power	1.00	0.35	0.33	2.29	0.38	0.116112164
Liquid Entry pressure	2.88	1.00	2.00	0.50	0.44	0.204823141
Rejection Rate	3.00	0.50	1.00	2.88	0.50	0.205390803
Thermal Power	0.44	2.00	0.31	1.00	0.26	0.123864583
Flux	2.62	2.29	2.00	3.91	1.00	0.349809308

**Table 6:** Composite weightages of CIFs of MD by using AHP-MDM.

S.No	Main DOs	Weightages	CIF	Local Weights	Global Weights	Overall Ranking
1	Pumping Power	0.116112164	Flow Rate	0.4381	0.0509	7
			Feed Channel Geometry	0.1846	0.0214	20
			Pressure Difference	0.2677	0.0311	17
			Viscosity	0.1096	0.0127	23
2	Liquid Entry pressure	0.204823141	Hydrophobicity	0.4081	0.0836	2
			Material Of Membrane	0.2062	0.0422	12
			Pore size	0.2156	0.0442	10
			Feed Properties (concentration and Surface tension)	0.1701	0.0348	15
3	Rejection Rate	0.205390803	Feed Concentration	0.2554	0.0525	6
			Pore size	0.2229	0.0458	9
			Type of Membrane	0.1064	0.0219	19
			Feed Temperature	0.4153	0.0853	1
4	Thermal Power	0.123864583	Temperature Difference	0.3212	0.0398	13
			Flow rate	0.5228	0.0648	5
			Specific Heat	0.1560	0.0193	22
5	Flux	0.349809308	Feed Temperature	0.1882	0.0658	4
			Flow Rate	0.2034	0.0711	3
			Feed concentration	0.1365	0.0477	8
			Duration	0.0602	0.0211	21
			Membrane Configuration	0.0841	0.0294	18
			Feed channel Geometry	0.0962	0.0337	16
			Membrane thickness	0.1102	0.0385	14
			Pore Size	0.1213	0.0424	11

**Table 7:** Pairwise comparison of derivable outputs of MD using Fuzzy-AHP.

	Pumping Power	Liquid Entry Pressure	Rejection Rate	Thermal Power	Flux	Weightages
Pumping Power	(1.00,1.00,1.00)	(0.25,0.33,0.50)	(0.25,0.33,0.50)	(1.00,2.00,3.00)	(0.25,0.33,0.50)	0.1050
Liquid Entry pressure	(2.00,3.00,4.00)	(1.00,1.00,1.00)	(1.00,2.00,3.00)	(0.33,0.50,1.00)	(0.33,0.50,1.00)	0.2001
Rejection Rate	(2.00,3.00,4.00)	(0.33,0.50,1.00)	(1.00,1.00,1.00)	(3.00,4.00,5.00)	(0.33,0.50,1.00)	0.2276
Thermal Power	(0.33,0.50,1.00)	(1.00,2.00,3.00)	(0.20,0.25,0.33)	(1.00,1.00,1.00)	(0.20,0.25,0.33)	0.1022
Flux	(2.00,3.00,4.00)	(1.00,2.00,3.00)	(1.00,2.00,3.00)	(3.00,4.00,5.00)	(1.00,1.00,1.00)	0.3651

**Table 8:** Composite weightages of CIFs of MD by using Fuzzy-AHP.

S.No	Main DOs	Weightages	CIFs	Local Weights	Global Weights	Overall Ranking
1	Pumping Power	0.1050	Flow Rate	0.4309	0.0452	10
			Feed Channel Geometry	0.1497	0.0157	21
			Pressure Difference	0.2914	0.0306	15
			Viscosity	0.1280	0.0134	23
2	Liquid Entry pressure	0.2001	Hydrophobicity	0.4085	0.0817	3
			Material of Membrane	0.2062	0.0413	12
			Pore size	0.2420	0.0484	8
			Feed Properties (concentration and Surface tension)	0.1432	0.0287	16
3	Rejection Rate	0.2276	Feed Concentration	0.2693	0.0613	6
			Pore size	0.2203	0.0501	7
			Type of Membrane	0.1131	0.0257	17
			Feed Temperature	0.3973	0.0904	2
4	Thermal Power	0.1022	Temperature Difference	0.2415	0.0247	18
			Flow rate	0.6131	0.0627	5
			Specific Heat	0.1454	0.0149	22
5	Flux	0.3651	Feed Temperature	0.1928	0.0704	4
			Flow Rate	0.2568	0.0938	1
			Feed concentration	0.1310	0.0478	9
			Duration	0.0562	0.0205	20
			Membrane Configuration	0.0675	0.0246	19
			Feed channel Geometry	0.0915	0.0334	13
			Membrane thickness	0.0847	0.0309	14
			Pore Size	0.1195	0.0436	11

**Table 9:** Weightages and ranks of CIFs using AHP-MDM and Fuzzy-AHP.

CIF	AHP-MDM	AHP-MDM RANKS	AHP-MDM Classification	Fuzzy-AHP	Fuzzy-AHP RANKS	Fuzzy AHP Classification
Flow Rate	0.1868	1	***	0.2017	1	***
Feed Temperature	0.1511	2	***	0.1608	2	***
Pore size	0.1324	3	***	0.1422	3	***
Feed Concentration	0.1002	4	***	0.1091	4	***
Hydrophobicity	0.0836	5	***	0.0817	5	***
Feed Channel Geometry	0.0551	6	***	0.0491	6	***
Material of Membrane	0.0422	7	**	0.0413	7	**
Temperature Difference	0.0398	8	**	0.0247	12	*
Membrane thickness	0.0385	9	**	0.0309	8	**
Feed Properties (concentration and Surface tension)	0.0348	10	**	0.0287	10	**
Pressure Difference	0.0311	11	**	0.0306	9	**
Membrane Configuration	0.0294	12	*	0.0246	13	*
Type of Membrane	0.0219	13	*	0.0257	11	**
Duration	0.0211	14	*	0.0205	14	*
Specific Heat	0.0193	15	*	0.0149	15	*
Viscosity	0.0127	16	*	0.0134	16	*

*** Extremely Important. ** Moderately Important. * Least Important.

## Supplementary Materials

The following are available online at https://www.mdpi.com/2077-0375/11/3/164/s1, Table S1: Crisp numeric value of Saaty’s nine-point scale [19]; Table S2: Triangular fuzzy scale for fuzzy analytic hierarchy process; Figure S1: Fuzzy Triangular number (A); Figure S2: Intersection of two fuzzy triangular numbers.
