# Supplementary material for: Evaluating Critical Influencing Factors of Desalination by Membrane Distillation Process—Using Multi-Criteria Decision-Making
Source: Membranes (Basel). 2021 Feb 27;11(3):164. doi: 10.3390/membranes11030164 (PMC7996794; doi:10.3390/membranes11030164)
Supplement: Supplementary file 1 [file membranes-11-00164-s001.pdf]

membranes

Supplementary Materials

Evaluating Critical Influencing Factors of Desalination by Membrane Distillation Process–Using Multi-Criteria Decision Making

Ali E. Anqi *, Azam A. Mohammed

Department of Mechanical Engineering, College of Engineering, King Khalid University, Abha 61421, Saudi Arabia
* Correspondence: aanqi@kku.edu.sa

Table S1: Crisp numeric value of Saaty's nine-point scale [19].

Linguistic scale	Numeric Value	Reciprocal Numeric value
Equally preferred	1	1
Moderately preferred	3	1/3
Essentially preferred	5	1/5
Very preferred	7	1/7
Extremely preferred	9	1/9
Intermediate importance between two adjacent judgments	2, 4, 6, 8	1/2, 1/4, 1/6, 1/8

Table S2: Triangular fuzzy scale for fuzzy analytic hierarchy process.

**Fuzzified linguistic scale**	**Triangular fuzzified numeric values**	**Reciprocal of triangular fuzzified Numeric value**
Equally preferred	(1,1,1)	$\left(\frac{1}{1},\frac{1}{1},\frac{1}{1}\right)$
Moderately preferred	(2,3,4)	$\left(\frac{1}{4},\frac{1}{3},\frac{1}{2}\right)$
Essentially preferred	(4,5,6)	$\left(\frac{1}{6},\frac{1}{5},\frac{1}{4}\right)$
Very preferred	(6,7,8)	$\left(\frac{1}{8},\frac{1}{7},\frac{1}{6}\right)$
Extremely preferred	(9,9,9)	$\left(\frac{1}{9},\frac{1}{9},\frac{1}{9}\right)$
Intermediate importance between two adjacent judgments	(1,2,3), (3,4,5), (5,6,7), (7,8,9)	$\left(\frac{1}{3},\frac{1}{2},\frac{1}{1}\right)$, $\left(\frac{1}{5},\frac{1}{4},\frac{1}{3}\right)$, $\left(\frac{1}{7},\frac{1}{6},\frac{1}{5}\right)$, $\left(\frac{1}{9},\frac{1}{8},\frac{1}{7}\right)$

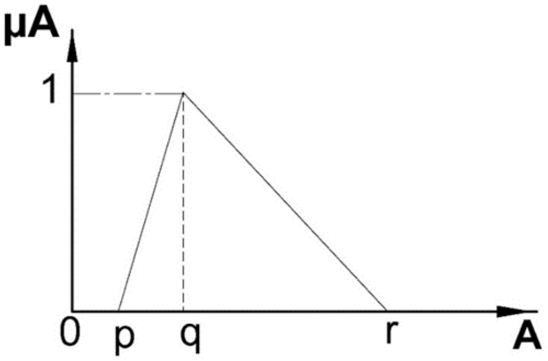

Figure S1: Fuzzy Triangular number (A).

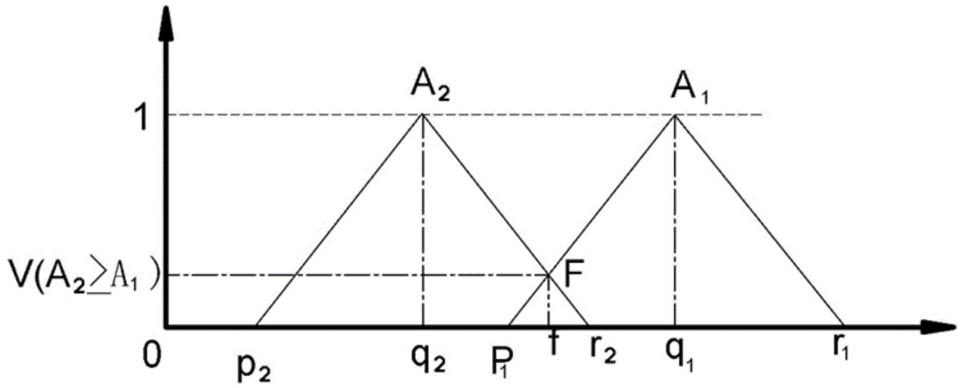

Figure S2: Intersection of two fuzzy triangular numbers.
